# Advances in Techniques in Radical Prostatectomy

**DOI:** 10.3390/medicina61071222

**Published:** 2025-07-04

**Authors:** Hui Miin Lau, Liang G. Qu, Dixon T. S. Woon

**Affiliations:** 1 Department of Urology, Peninsula Health, Frankston, VIC 3199, Australia; 2 Department of Surgery, University of Melbourne, Melbourne, VIC 3010, Australia; 3 Department of Urology, Austin Health, Heidelberg, VIC 3084, Australia

**Keywords:** prostate cancer, radical prostatectomy

## Abstract

Since its development in 1904, radical prostatectomy (RP) has remained a fundamental surgical option in the management of localised prostate cancer. Over time, continuous advancements in surgical techniques have improved oncological outcomes while reducing functional complications. This narrative review explores the evolution of RP, depicting its progression from the traditional open approach to minimally invasive laparoscopic and robotic-assisted techniques. Key developments in RP techniques, including nerve-sparing, bladder neck-sparing and Retzius-sparing techniques as well as enhanced perioperative management, have contributed to reduced postoperative complications, namely incontinence and erectile dysfunction. Additionally, technological innovations such as augmented reality, utilising indocyanine green for improved visualisation of prostatic boundaries and illuminare-1 to easily identify nerves intraoperatively, artificial intelligence, and novel molecular imaging technologies such as PSMA PETs for improved margin assessment are shaping the future of RPs. Despite these advancements, challenges persist, including a steep learning curve associated with newer techniques, disparities in access due to cost considerations, and a lack of standardised outcome measures across different surgical approaches. This review provides insight into current trends, ongoing challenges, and future directions that may further refine surgical precision, enhance patient safety, and improve long-term treatment success in prostate cancer management.

## 1. Introduction

Prostate cancer is the fourth most common cancer worldwide and the second most common cancer in men. In 2022 alone, 1,467,854 new cases of prostate cancer and 397,430 deaths were reported [1]. France had the highest age-standardised rate of 157.5, followed by Lithuania with 135, while the United States of America (USA) had the highest incidence, contributing 15.7% of total new cases (230,125/1,467,854), followed by China making up 9.14% of the new cases (134,156/1,467,854) [1].

Treatment options for localised prostate cancer currently include active surveillance, curative external beam radiation therapy (EBRT), brachytherapy, and radical prostatectomy (RP). Active surveillance may be offered to low- and select intermediate-risk patients to minimise treatment-related toxicity without compromising survival. The standard treatment approach for EBRT may involve intensity-modulated or volumetrically modulated arc therapy with image-guided radiotherapy, often combined with androgen deprivation therapy. RP involves the removal of the entire prostate gland with the purpose of removing all prostate cancer while simultaneously preserving pelvic organ function. Each treatment approach is tailored to the patient’s risk profile, cancer stage and personal preference with the goal of optimising outcomes while minimising side effects.

This review will detail the traditional techniques in RP, advances in Robot-Assisted Radical Prostatectomy (RARP), the surgical techniques and approaches to RP, long-term outcomes and the future directions and emerging technologies for RP. Relevant studies were identified through PubMed and Cochrane searches of English-language articles published between 2010 and 2025 with a preference for systematic reviews and meta-analysis, prioritising those with larger sample sizes and longer follow-up periods to enhance the reliability and ensure a balanced representation of current evidence.

## 2. Traditional Techniques in Radical Prostatectomy

### 2.1. Open Radical Prostatectomy

There are various approaches to RP, with retropubic being the most common surgical approach used. Historically, RP was first described using a perineal approach at The Johns Hopkins Hospital in 1904 by Young [2]; however, in 1945 the retropubic approach was introduced by Millin at the All Saints Hospital in London [3].

The technique of retropubic RP is classically described by firstly positioning the patient supine with the pubis over the break. A midline skin incision is performed through subcutaneous tissues and fascia incised. A retractor is used to ensure adequate exposure; the prevesical space is opened and endopelvic fascia is divided. Both the superficial and deep dorsal vascular complex (DVC) isolated are ligated. A fasciotomy is made in the groove between the neurovascular bundles (NVBs) and the prostate and is extended. Once the NVB has been completely released anteriorly, Denonvilliers’ fascia is sharply incised allowing for complete lateral release. The apex of the prostate can then be mobilised, and the anterior two-thirds of the urethra is divided. Urethral anastomoses are created by placing sutures at 9, 11, 1 and 3 o’clock positions, followed by posterior sutures at 5 and 7 o’clock positions. As the prostate is mobilised off the rectum, the vascular pedicle is carefully identified, clipped, and divided, allowing the NVB to separate from the prostate fully. The pedicles must be isolated and ligated to expose the seminal vesicles and fully mobilise the prostate. The bladder neck (BN) is then incised anteriorly over the Foley catheter balloon, followed by circumferential incision of the BN to free the specimen. The mucosa may be everted followed by completion of the vesicourethral anastomosis. A new urethral catheter is passed before the anastomotic sutures are securely tied down.

Radical perineal prostatectomy is infrequently performed nowadays though it remains historically significant. Its falling out of favour is primarily due to the difficulty in nerve sparing and lymph node removal. The described surgical technique involves positioning the patient in lithotomy and performing an incision around the anal rim. Subcutaneous fascia and fat tissue are divided, the central tendon securing the anal sphincter is transected and the ischiorectal fossa is developed bluntly. The levator ani are retracted laterally and rectourethralis is dissected. The rectum is mobilised and retracted downward and a curved Lowsley retractor placed into the bladder brings the prostate to the perineal plane. The white posterior layer of Denonvillier’s fascia is incised before the prostatic pedicles are dissected and the seminal vesicles are mobilised. The dorsal veins and puboprostatic ligaments are bluntly separated from the apex. The urethra is dissected, and usually four to five sutures are placed at the stump. The posterolateral surfaces of the prostate are bluntly separated from the bladder base and neck up to the proximal urethra, which can be preserved for anastomosis. The prostate with seminal vesicles is excised. A vesico-urethral anastomosis incorporating the recto-urethralis muscle is performed. A drain is placed, the levator ani are re-approximated without tension, and the wound is closed [4].

### 2.2. Laparoscopic Radical Prostatectomy

Minimally invasive laparoscopically assisted radical prostatectomy (LAP) was first performed in 1991. Early case series demonstrated equivalence to the open approach with respect to tumour removal, continency, and potency [5]. However, the laparoscopic approach was associated with prolonged operative times, averaging 9.4 h, and presented significant technical challenges. Therefore, at the time of its introduction, LAP was not considered a viable surgical alternative to the open approach for the treatment of prostate cancer.

Since then, several different approaches have been described, namely the transperitoneal (TP) and extraperitoneal methods. The TP approach offers maximum mobility of the bladder, facilitating a tension-free vesicourethral anastomosis. Additionally, the intra-abdominal cavity provides an expansive surgical field, enhancing instrument manoeuvrability. These advantages contributed to the TP approach becoming the predominant minimally invasive technique. However, TP prostatectomy presented other difficulties: direct intra-abdominal communication required bowel manipulation, potential for postoperative ileus, intraperitoneal communication of blood and urine, and potential damage to organs otherwise not involved. These complications led to refinement and development of an extraperitoneal approach [6].

The extraperitoneal approach was first described by Raboy et al. in 1997 [7]. Initial case series reported significant reductions in operative time, with an estimated blood loss of 600 cc and average hospital stay of 2.5 days [7]. Furthermore, the extraperitoneal approach requires less Trendelenburg positioning, reducing the need for bowel retraction and resulting in improved anaesthetic and cardiovascular parameters. However, the main limitation of this approach is the restricted working space, which may be mitigated with increased surgical experience [6].

## 3. Advances in Robot-Assisted Radical Prostatectomy

The first RARPs were performed in Germany in 2000 [8] but did not garner interest in the USA until 2002 when Menon et al. [9] compared results of RARP with open radical prostatectomy (ORP) and reported that the robotic technique had reduced blood loss, contributing to its early and rapid adoption thereafter. Menon et al. [10] later developed the standardised Vattikuti technique of RARP, reporting favourable outcomes, both oncologically and functionally. This led to a surge in the application of RARP worldwide, first in the USA, subsequently in Europe, and later worldwide [11].

### 3.1. Technology and Innovation in RARP

Robotic surgical systems provide enhanced precision and dexterity, significantly benefiting surgeons by improving accuracy and control during procedures [12]. The utilisation of robotic arms for positioning and holding surgical instruments both alleviates the physical burden on surgical assistants and reduces cognitive strain on surgeons by ensuring superior instrument stability and precision, ultimately enhancing patient safety and postoperative outcomes [13].

Advancements in robotic surgery have incorporated biocompatible soft materials, superelastic alloys, and 3D-printed polymers, such as silicone elastomers, which enable robots to adapt their shape and mechanical properties in response to external forces, thereby increasing intrinsic safety [14]. Furthermore, recent innovations emphasise enhanced flexibility, bendability and stiffness control, allowing precise instrument positioning. The force exerted during shape modulation is monitored, with stiffness adjustments at the instrument tip controlled via tension, facilitating active adjustment during procedures [15]. In addition, the robot-assisted surgical system offers a stable camera platform, high-definition binocular vision of the surgical field, and visual magnification, which result in better surgical outcomes and reduced fatigue, in comparison to a 2D laparoscopic view with limited depth perception [12].

### 3.2. Challenges and Criticisms

The benefits of RARP are accompanied by significant challenges, primarily due to a steep learning curve. Although acceptable operative times can be achieved in less than 20 cases, positive surgical margin (PSM) rates may not plateau until after performing more than 80 cases [16]. Moreover, robotic assistance does not mitigate the challenges associated with obese patients, those with large prostates or middle lobes, or individuals with previous surgeries. In these cases, clinical outcomes tend to be less favourable. In addition, there are significant economic barriers preventing the widespread adoption of robotic technology [16]. The implementation costs of robot-assisted surgery are substantial, with the acquisition of the da Vinci Xi, along with sterilisation equipment and theatre refurbishment, exceeding USD 4 million. Ongoing maintenance further exacerbates the financial burden [17]. Furthermore, for two decades, the da Vinci was the only available robotic platform before new platforms such as the Hugo RAS, Versius, and Senhance were introduced and proven to achieve comparable results. Due to these high costs, its use is limited in Africa, South America and parts of Asia [18].

### 3.3. Benefits and Outcomes of Minimally Invasive Surgery

**Figure 1:**
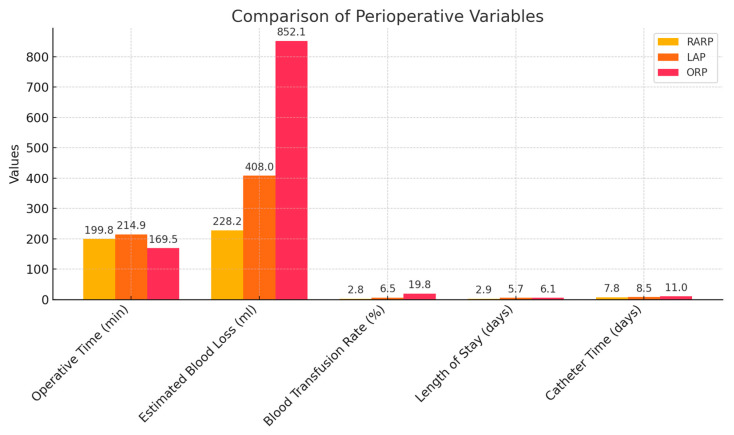
Graphical representation of comparisons of perioperative variables in RARP vs. LAP vs. ORP.

A systematic review involving 1,353,485 patients across 559 studies on ORP, 413 on LAP and 752 on RARP revealed that minimally invasive surgery yielded the best perioperative and complication results, especially RARP, which was associated with less complex cases, higher annual surgeon volume, and greater performance [19]. Tewari et al. [20] compared patient-reported pain after RARP and ORP and found that those who underwent RARP reported less pain. Similarly, other centres reported less analgesic use after LAP compared with ORP. To achieve a complication rate of 12.3%, which was the average for RARP, surgeons needed an annual volume of 30, 95 and 95 procedures per surgeon per year for RARP, LAP, and ORP, respectively [19]. RARP showed better performance for all perioperative variables studied except for operative time (as shown in Figure 1). Minimally invasive surgical techniques also showed lower incidence of bladder neck contracture rates from 0 to 5.8% compared to 3–14% in ORP [21,22,23]. Despite these benefits, the availability of RARP remains limited by the adoption of robotic surgical facilities and by surgeon training, and laparoscopy remains an alternative in places with no access to robotic surgery [24]. Additionally, in the hands of experienced surgeons, ORP continues to be a widely practiced and viable approach to RP.

For comparison between minimally invasive techniques, a study was conducted in a single centre on 327 patients, 156 who underwent RARP and 171 LAP, complication rates were comparable, although patients who underwent LAP experienced lower median bleeding (250 vs. 719.5 mL) and shorter hospitalisation time. Functional outcomes were better for LAP over 18 months, while oncological outcomes over 36 months were not significantly different between arms [25].

## 4. Surgical Techniques and Approaches

### 4.1. Nerve-Sparing Techniques

Nerve-sparing (NS) techniques can be categorised based on thermal use (athermal vs. thermal), extent (unilateral vs. bilateral), fascial planes of dissection (intrafascial, interfascial or extrafascial), and direction of dissection (antegrade or retrograde). The Veil of Aphrodite is an athermal technique where dissection begins between the prostate fascia and lateral pelvic fascia from the base of the seminal vesicles, proceeding along interfascial planes, suspending the periprostatic tissue with NVB from the pubourethral ligaments [26]. In contrast, thermal techniques include pre-emptive local hypothermia to the pelvis using an endorectal cooling balloon system (ECB) to decrease the metabolic rate and reduce inflammation. Additionally, a CO_2_ laser has also been explored for NVB dissection [27].

The impact of unilateral versus bilateral NS techniques remains debated: Finley et al. [28] and Nilsson et al. [29] found no notable difference in outcomes. Conversely, the CEASAR study observed better potency and continence outcomes after bilateral NS [30]. NS techniques also vary based on the fascial plane of dissection. In extrafascial NS, dissection is performed under Denonvilliers’ fascia. Interfascial NS preserves the prostatic fascia, with dissection occurring between the prostatic fascia and the lateral pelvic fascia. Intrafascial NS is a more refined technique preserving supplementary nerve fibres on the anterolateral surface of the prostate by dissecting between the prostatic capsule and fascia [31].

Lastly, NS techniques can be divided based on the direction of dissection. Early retrograde release of the NVB begins with identifying the landmark prostatic artery and developing a plane between the artery and the prostate, continuing posteriorly. The dissection proceeds in a retrograde way towards the base of the prostate. Antegrade NS is performed from the prostate base towards the apex with the vascular pedicles transected first. Gentle upward traction is used to identify the prostatic pedicles and counter traction of the prostate exposes the space between the lateral pelvic fascia, Denonvilliers’ fascia and the prostatic fascia before either interfascial or intrafascial dissection is carried out [28]. Despite advancements in NS techniques, functional outcomes are influenced by preoperative potency, tumour extent and, most importantly, patient anatomy. Limited evidence proves any approach superior, highlighting the need for individualised surgical strategies [32] (Table 1).

### 4.2. Bladder Neck Preservation and Reconstruction Techniques

Bladder neck-sparing (BNS) techniques were first reported in 1993 to protect the sphincter to avoid incontinence [33]. Traditionally, the described technique involved dissection through the connective tissue of the detrusor apron midline at the vesico-prostatic junction until bladder fibres are seen. The incision is extended lateral in an arced fashion, followed by blunt dissection in a caudal direction over the anterior BN, identifying the vertical fibres of the prostatic urethra. The BN is opened anteriorly, and the urethral catheter is withdrawn. The posterior bladder mucosa is then incised, and the dissection is continued posteriorly and laterally until adipose tissue is encountered lateral to the BN [34].

The use of BNS in open, laparoscopic and robot-assisted RP has been compared in previous reviews. Most studies reported no significant difference in oncological outcomes using a BNS or BN reconstruction technique. Early urinary continence (UC) rates ranged from 36% to 100% at 1 month, with the long-term UC rate reported at 84–100% at 12 months if the BN was spared. BNS may improve early return and long-term UC without compromising oncological outcomes. The anastomotic stricture rate may also be lower when adopting the BNS technique, ranging from 1 to 5% compared to 11–18% with BN reconstruction techniques [35].

### 4.3. Retzius-Sparing Approach

A Retzius-sparing (RS) approach has also been developed for RARP, to spare the anterior support structures that may preserve functional outcomes. A posterior approach is undertaken, where the parietal peritoneum is incised at the level of the pouch of Douglas, and the seminal vesicles are identified and retracted. Posterior prostatic dissection is performed in an antegrade fashion. Thereafter, the BN is incised laterally, and finally, anterior prostatic dissection is carried out, also in an antegrade fashion. A systematic review compared the different approaches used to perform RARP (anterior, transperitoneal RARP and RS-RARP) and the UC rates at different time points postoperatively (1 month, 3 months, 6 months and 12 months). UC rates were significantly higher in favour of the RS approach at 1 and 3 months but there was no significant advantage in the long term [36].

## 5. Postoperative Complications and Management

### 5.1. Post-Prostatectomy Urinary Incontinence

Post-prostatectomy urinary incontinence (UI) is common and can be mitigated with guided pelvic floor muscle training [37]. Quantifying the degree of urine leakage using incontinence pad use is crucial to determine whether subsequent surgical treatments are required: implantable slings are effective in men with mild-to-moderate UI while implantation of an artificial urinary sphincter (AUS) remains the gold standard in men with severe UI or a history of radiation exposure. In a meta-analysis to assess the efficacy of the male sling and AUS on post-prostatectomy UI, both the sling and AUS significantly reduced the number of pads used per day by about three and increased the quality of life compared with before intervention [38].

The sling procedure has benefits of being a lower-risk and less-invasive approach, especially for those wishing to avoid use of a mechanical device. Adjustable slings make postoperative tension adjustments possible and have been tested in a patient population with severe UI, with some systems reporting good results in irradiated patients. In comparison, the AdVance™ XP sling (Boston Scientific, Marlborough, MA, USA) has an easy implantation technique and a well-defined patient profile (mild UI, no irradiation, positive urethral repositioning test). However, postoperative tension adjustments are difficult and there may be a high removal rate (up to 35%) [39]. Adequate urethral tissue compliance is necessary for successful compression and/or proximal repositioning of the urethra with a sling. Radiation exposure or previous AUS explantation may result in a relatively noncompressible urethra and diminished sling effectiveness [40].

In comparison, experiences with the AUS report overall success rates (usually defined as 0–1 pads per day) over 80%. A surgical revision rate of 13–43% should be noted, however [39]. AUS is more invasive, and implantation carries a well-known risk of revision surgery secondary to infection, erosion, urethral atrophy and mechanical failure. Walsh et al. [41] reported a revision rate due to urethral atrophy, infection and erosion after radiotherapy of 41% (vs. 11% in patients without irradiation) after 46 months in 98 patients.

### 5.2. Erectile Dysfunction

The first-line treatment in post-RP erectile dysfunction (ED) is phosphodiesterase type 5 inhibitors (PDE5i) due to their ease of use, safe profile, and positive effect on erectile function. This is often coupled with vacuum erection devices, followed by intracavernosal injections. The penile prosthesis implant is regarded as a third-line treatment for patients with ED. A review of 39 RCTs concluded that PDE5i remains the first-line treatment for patients with ED after RP. Regular and on-demand PDE5i had a much higher mean erectile function domain of the ‘International Index of Erectile Function’ scores within 3 months after RP in comparison to placebos [42]. Intracavernosal injections may also lead to very high response rates of greater than 70%. Injections do however subject patients to side effects of pain in 13–33%, fibrotic complication in 1–57%, and priapism in 5–23% of patients [43].

Various penile prosthesis devices are available for managing post-prostatectomy ED, restoring sexual function and improving quality of life. Malleable devices feature two solid intracavernosal implants, bent upward for erection simulation or downward when not in use. Advantages include simplified surgical implantation, ease of use, and lower failure rates. However, erosion rates may be higher [44]. The current gold standard for penile prostheses is the three-piece inflatable model, comprising 90% of new implants [45]. It consists of two cylinders in the corporal bodies, a fluid reservoir placed submuscularly or within the space of Retzius, and a scrotal pump for inflation control [46]. Alternatively, a two-piece inflatable prosthesis with a scrotal pump and smaller reservoirs is useful in patients with previous pelvic surgery or a history of pelvic radiation, as it avoids the need for dissection within the abdomen or pelvis [47]. Patient satisfaction rates exceeded 80% and 70% for three-piece and two-piece inflatable models, respectively [44].

## 6. Long-Term Outcomes and Quality of Life

### 6.1. Functional Outcomes: Continence and Sexual Function

Functional outcomes post-RP are primarily assessed based on UI, defined as the use of at least one protective pad used in a 24 h period, and ED, an erection insufficient for intercourse more than half of the time. In a Swedish trial, LAPPRO, robotic prostatectomy was compared to the open procedure. At 24 months, a significant difference in ED was seen in favour of RARP (68% vs. 74%) but no significant difference was observed for UI (19% vs. 16) [48]. With regard to long-term outcomes, UI was no different at 8 years after surgery between RARP and ORP (27% vs. 29%) while ED was significantly lower in the RARP group (66% vs. 70%) [49].

In a study based in Australia, of 3826 patients undergoing RP, 1047 received ORP and 2779 received RARP. There were no statistically significant differences for RARPs, compared to ORPs, in terms of UI, urinary irritative/obstructive domain scores, pad usage and ED. RARP patients had slightly higher sexual domain scores. There were no differences in urinary patient-reported outcomes between ORP and RARP when assessed 12 months post-surgery. The sexual domain slightly favoured RARP, but this was not deemed clinically significant [50].

### 6.2. Oncological Outcomes

Recent studies have compared RARP with ORP in terms of oncological outcomes, including PSM, biochemical recurrence, and prostate cancer-specific mortality (PCSM). Across these outcomes, RARP demonstrated at least equivalent if not superior outcomes compared to ORP, particularly in reducing PSM rates and improving long-term survival. Despite variations in study designs, follow-up periods, and patient populations, the findings consistently suggest that RARP offers advantages, especially in high-volume institutions and patients with organ-confined disease. However, further research is required to confirm these results and eliminate confounding factors.

A study conducted in Australia with 12,394 patients found that the proportion of PSM in pT3/4 disease declined from 50% to 38% in 2011–12 to 2019–20, while pT2 percentages were consistent at 13%. High-volume institutions demonstrated a fall in pT2 PSM from 12% to 6.5%. Independent predictors of lower PSM were robotic versus open method and being treated at a private versus public institution. A clear decline in the proportion of pT3 PSM was observed in a large prostate cancer registry. Possible explanations include improved proficiency with robotic surgery and participation in a registry-based quality improvement initiative [51].

In an extended Swedish study with an 8-year follow-up, PCSM was significantly lower in the RARP group 8 years after surgery (40/2699 vs. 25/885). These differences were mainly seen in the group with high D’Amico risk, with a lower risk of PSMs (21% vs. 34%), biochemical recurrence (51% vs. 69%), and PCSM (14/220 vs. 11/77) for RARP versus ORP. A relationship between surgical technique and mortality cannot be inferred, but the result demonstrates that RARP is oncologically safe [49]. Overall, the studies suggest that RARP may reduce PSMs and improve oncological outcomes compared to ORP, especially for high-risk patients, although further randomised studies are needed to confirm these findings.

## 7. Future Directions and Emerging Technologies

### 7.1. Artificial Intelligence

A study introduced a deep learning method for the assessment of prostatectomy and proposed additional factors to assess the impact of the relevant anatomical relationships between the prostate and pelvis on surgical difficulty. The two-stage deep learning method for landmark localisation can be integrated into surgical navigation systems, facilitating more precise localisation of the prostate and reducing damage to healthy tissue. Spatial relationships and metrics from pelvic floor artificial intelligence (AI) analysis can inform personalised surgical approaches, ensuring that the surgical strategy aligns with the patient’s unique anatomy and musculature. In clinical practice, these metrics can be incorporated into decision-support tools for personalised risk assessment [52].

AI has also been widely used in predictive analyses for outcomes. In an analysis of a cohort of 8524 patients, two separate machine learning Artificial Neural Network models were trained to predict functional outcomes (continence and potency) at 12 months post-RP. They demonstrated the ability to effectively predict functional outcomes and outperformed other algorithms. However, optimising models and investigating other factors are required to improve predictive accuracy and clinical applicability. Multi-centre studies and larger datasets will further contribute to enhancing the value of AI in clinical decision-making for prostate cancer treatment [53]. Another powerful AI tool for improving proficiency, anatomical comprehension, and patient outcomes through more precise, standardised training is gesture classification. When integrated with video analysis, it promotes active learning where surgeons can receive immediate feedback, review their performance and refine each gesture. This breakdown of surgeries into gestures also acts as a potential platform for future automation [54].

### 7.2. Augmented Reality in Surgery/Molecular Imaging and Targeted Therapies

The recent adoption of indocyanine green (ICG) fluorescence in surgery has led to its exploration for use in RP. The use of ICG for assessing diagnostic performance of pelvic sentinel lymph node fluorescence showed relatively unsatisfactory results in a systematic review [55]. However, in another study evaluating ICG-assisted NS-RARP, ICG improved the visualisation of prostatic boundaries and showed significant improvements in lower urinary tract symptoms without affecting operative time or increasing adverse events [56]. This suggests that ICG may have a role in assisting NS techniques in RARP.

Another promising technology is illuminare-1, which binds to myelin, an insulating sheath around the nerves. When a blue light is shone, the agent glows, increasing the visibility of the nerves. A recent clinical trial assessed its feasibility in patients receiving illuminare-1 by intravenous infusion early in the operation. Within a short time, the nerves were fully illuminated and remained brightened throughout surgery [57]. This may have further applications in offering more precision in NS aspects of RP.

Additionally, a study evaluating the accuracy of functional tumour-volume segmentation in intraoperative ex vivo PET/CT for margin assessment during RP showed favourable results. Seven high-risk prostate cancer patients received [18F]PSMA-1007 before RP. Post-RP, ex vivo imaging assessed functional tumour volume. Resection margins and volumes were compared with histopathology. Sensitivity, specificity, and positive and negative predictive values of margin detection were 83%, 100%, 100%, and 92%, respectively. Intraoperative ex vivo PET/CT is a promising tool for margin assessment, with likely applications for patients with high-risk prostate cancer [58].

Another promising technology is neurovascular structure-adjacent frozen-section examination (NeuroSAFE), an intraoperative frozen-section examination looking at surface margins for cancer cells and, if detected, further removal can be undertaken immediately. Frozen sections are taken intraoperatively and examined by a pathologist to look for cancer cells. In a systematic review and meta-analysis, a total of 4207 patients were analysed: 2247 undergoing RARP with NeuroSAFE and 1960 without. There was a great reduction in the likelihood of PSM at final pathology and biochemical recurrence in favour of NeuroSAFE. This procedure also led to greater UC and erectile function [59].

Lastly, the Levita^®^ Magnetics Magnetic-Assisted Robotic Surgery (MARS) platform is an innovative surgical system that utilises magnetic retraction. It makes use of internal magnetic graspers controlled externally, minimising the number of incisions required and the need for surgical assistants, allowing surgeons more precise, autonomous control of instruments [60].

### 7.3. Single-Port Robot-Assisted Radical Prostatectomy

Single-Port Robot-Assisted Radical Prostatectomy (SP-RARP) employs a single incision with various techniques—transperitoneal, extraperitoneal, transvesical, and transperineal—having been safely and feasibly trialled using the da Vinci SP^®^ system. The advantages of SP-RARP include superior cosmetic outcome and reduced psychosocial impact. Furthermore, with direct percutaneous entry into the bladder, transvesical SP-RARP eliminated the need for a steep Trendelenburg position. The supine positioning may also apply to other SP-RARP approaches, offering the potential to perform RARP under regional anaesthesia [61]. The use of epidural anaesthesia has proven successful in 12 patients who underwent SP-RARP with no anaesthetic-related complications [62].

Similar outcomes have been observed for SP-RARP and multiport RARP in terms of operative time, catheterisation time, pain scores, complication rates, continence and potency rates, PSM, and biochemical recurrence. SP-RARP exhibited shorter LOS and lower incidence of UI when compared to other techniques, while no differences were reported in terms of postoperative International Prostate Symptom Score, post-void residual or maximum urinary flow rate. Overall, the SP approach offers comparable oncological, functional, and perioperative outcomes [63].

### 7.4. Investigation of Same-Day Discharge

Over the last decade, there has been increasing evidence supporting the safety of same-day discharge (SDD) after RARP. There are similar complication rates, readmission rates, and emergency department visits compared to inpatient stays, and SDD may significantly reduce healthcare costs. Savings of up to USD 2106 (19%) per patient may be seen with SDD compared to inpatient stays [64,65]. However, SDD is not feasible in all patients such as those with comorbidities, bleeding disorders, high body mass index, or salvage surgery, those living far from the hospital, and those with unavailability of a caretaker at home, as these patients require additional monitoring post-surgery. These might include more regular vital signs or blood tests the following day. Despite the advantages and preparations performed for SDD, 56% of patients are not discharged on the day of surgery due to intractable pain refractory to oral analgesics, catheter discomfort (45%), and postoperative nausea/vomiting (15%) [66]. Currently, the evidence on SDD is mostly observational, with no randomised controlled trials. Most studies come from high-volume centres, and the adoption of SDD in community hospitals is still limited. Additionally, there is no consensus on how to define “SDD failure,” which affects the interpretation of outcomes. Nevertheless, SDD after RARP shows promise with careful patient selection, adequate preparation, and standardised protocols. Further research is needed to better understand its applicability and to standardise reporting outcomes.

### 7.5. Telesurgery/Remote Surgery

Telesurgery enables surgeons to operate on patients remotely, offering the potential to overcome geographical boundaries in healthcare. Reliable connectivity is crucial to the safety of the patient, necessitating adequate technological infrastructure to support two-way transmission. Low-latency, high-bandwidth and secure connections are essential to ensure the success of the surgery. However, telesurgery presents ethical and legal concerns regarding informed consent and safeguarding patient data. Transmission of medical information and images across digital platforms increases risk of security threats and data breaches. Furthermore, the physical distance between surgeon and patient can hinder effective communication. Legal systems also leave unresolved issues surrounding liability in the event of technical failure. While telesurgery holds immense potential to extend surgical care to underserved or remote populations, its widespread adoption remains hindered by these technological, ethical, and legal concerns [67].

## 8. Conclusions

Since its introduction, RP has undergone significant technological advancements, greatly improving surgical outcomes. These innovations have not only reduced the duration of the procedure and the length of patients’ hospital stays but also enhanced the management of postoperative complications. Increasingly detailed research into prostate anatomy during RP has made it possible to preserve sexual function without compromising cancer control, and fewer patients experience issues with continence following prostatectomy. Robotic systems continue to evolve, offering surgeons enhanced precision and control, which minimises human error and improves their ability to navigate intricate anatomical areas. Additionally, emerging technologies like 3D imaging and augmented reality are providing surgeons with a more detailed view of the prostate and surrounding tissues. Challenges remain in ensuring widespread access to minimally invasive techniques. Many studies on the various newer techniques are limited by short follow-up periods and small sample sizes, making it difficult to draw definitive conclusions or make generalisable comparisons. Longitudinal studies with larger, more diverse patient populations are crucial to evaluate the long-term outcomes and benefits of these approaches. While there is a potential for selection bias in a narrative review, efforts were made to mitigate this by selecting articles with larger sample sizes and extended follow-up periods. All in all, the future of prostate cancer care remains promising, with continuous innovations driving progress in treatment options and patient care as well as continued improvement in patient outcomes.

## Figures and Tables

**Table 1:** Summary of various NS techniques and benefits of different RP techniques.

Nerve-Sparing Techniques		
Benefits: Better functional outcomes—individualised selection required depending on patient		
Thermal use	Athermal	Thermal
	Veil of Aphrodite	1. Endorectal Cooling Balloon system 2. CO_2_ laser
Extent	Unilateral vs. Bilateral	
Fascial Plane of Dissection	Intrafascial vs. Interfascial vs. Extrafascial	
Direction of Dissection	Antegrade vs. Retrograde	
Bladder Neck Preservation and Reconstruction Techniques		
Benefit: Improve early return and long-term UC rates		
Retzius-Sparing Approach		
Benefit: Spares the anterior support structures that may preserve functional outcomes		

## Data Availability

No new data were created or analysed in this study.

## Abbreviations

The following abbreviations are used in this manuscript:

AI	Artificial Intelligence
AUS	Artificial Urinary Sphincter
BN	Bladder Neck
BNS	Bladder Neck Sparing
DVC	Dorsal Vascular Complex
EBRT	External Beam Radiation Therapy
ECB	Endorectal Cooling Balloon
ICG	Indocyanine Green
LAP	Laparoscopically Assisted Radical Prostatectomy
LOS	Length of Stay
NeuroSAFE	Neurovascular structure-adjacent frozen-section examination
NS	Nerve-Sparing
NVB	Neurovascular Bundle
ORP	Open Radical Prostatectomy
PCSM	Prostate Cancer-Specific Mortality
POD	Postoperative Day
PSM	Positive Surgical Margin
RARP	Robot-Assisted Radical Prostatectomy
RP	Radical Prostatectomy
RS	Retzius-Sparing
SDD	Same-Day Discharge
SP	Single Port
TP	Transperitoneal
UC	Urinary Continence
UI	Urinary Incontinence
